# External Cervical Resorption Secondary to Orthodontic Treatment in a Previously Traumatized Tooth: A Case Report

**DOI:** 10.7759/cureus.106587

**Published:** 2026-04-07

**Authors:** Ahmed M Alghamdi, Abier F Alhekeir, Saad A Al-Nazhan

**Affiliations:** 1 Dentistry, Al-Iman General Hospital, Riyadh First Health Cluster, Riyadh, SAU; 2 Restorative Dentistry and Endodontics, Riyadh Elm University, Riyadh, SAU

**Keywords:** apicectomy, cone beam computed tomography, dental trauma, endodontic management, endodontics restorative dentistry, endodontic surgery, external cervical resorption, orthodontic treatment, radicular cyst, root resorption

## Abstract

A history of dental trauma and orthodontic treatment can directly or indirectly cause external root resorption (ERR), which may significantly compromise the structural integrity, pulp vitality, and periapical health of affected teeth. External cervical resorption (ECR) is an invasive form of ERR that originates at the cementoenamel junction and progresses three-dimensionally along the root surface.

In this case, a 20-year-old woman presented with discoloration of the maxillary right central incisor following orthodontic treatment, with a history of prior dental trauma. The maxillary right central and lateral incisors were diagnosed with pulp necrosis and asymptomatic apical periodontitis. Cone-beam computed tomography revealed a large periapical radiolucency associated with both teeth. Multiple forms of ERR were observed, including Patel Class 3Cp ECR on the palatal surface, extending into the interproximal area of the maxillary right central incisor.

After endodontic intervention, persistent symptoms necessitated apical surgery for enucleation of the periradicular pathology. Intentional replantation was considered but abandoned following intraoperative assessment. In the end, the affected tooth was extracted because of compromised structural integrity and poor periodontal support.

This case highlights that ECR may remain undetected and progress to advanced structural damage, with pulpal involvement and periradicular pathology, resulting in complex management and potential tooth loss.

## Introduction

Dental trauma can cause permanent damage to apical blood vessels, while orthodontic forces may compress the periodontal ligament (PDL), disrupting the vascular supply [1]. These changes increase a tooth’s susceptibility to pathological root resorption [2].

External root resorption (ERR) is classified based on location and etiology into surface, inflammatory, and cervical types [3]. External surface resorption (ESR) is typically a transient, self-limiting process triggered by mechanical stimuli, including pressure from adjacent pathology [3].

In contrast, external cervical resorption (ECR) is an invasive and progressive condition that originates on the root surface immediately below the epithelial attachment [4]. It progresses through three phases: initiation, resorption, and repair [2]. The process begins with damage to the protective cementum layer, exposing dentin to osteoclastic activity [5]. Unlike external inflammatory resorption (EIR), which is associated with infected necrotic pulps, ECR often occurs in teeth with vital pulps [1]. This is attributed to a pericanalar resorption-resistant layer of predentin that protects the root canal space [6]. Progression of ECR is influenced by factors such as localized hypoxia within the compressed PDL and microbial activity from the gingival sulcus or infected pulp [6,7].

The reported prevalence of ECR among patients undergoing endodontic evaluation is approximately 2.3% [8]. However, this may underestimate its true frequency, as many cases are detected incidentally [7]. Radiographic studies indicate that 70.2% of cases are identified during the active resorptive phase [7].

Accurate diagnosis is essential, as periapical radiographs may underestimate the true extent of ECR, correctly identifying affected root surfaces in 49.4% of cases [9]. Cone-beam computed tomography (CBCT) provides three-dimensional assessment and has been shown to alter the long-term prognosis in approximately 31.1% of cases, often shifting it from “good” to “poor” [9].

Management depends on lesion extent, location, and accessibility. Arrested lesions may be monitored, whereas progressive lesions require intervention [3]. Treatment options range from conservative restorative approaches to more complex internal, external, or combined repair strategies [3,10]. In advanced cases, intentional replantation (IR) may be considered; however, extraction remains the final option when structural integrity and periodontal support are severely compromised [3,10].

This report presents a case of advanced external cervical resorption in a previously traumatized tooth following orthodontic treatment. It demonstrates how the condition can progress silently until it results in tooth discoloration, extensive structural damage, and periradicular pathology. The case also highlights the diagnostic challenges and the complexity of management, where treatment options may ultimately be limited to extraction in advanced cases.

## Case presentation

**Figure 1:**
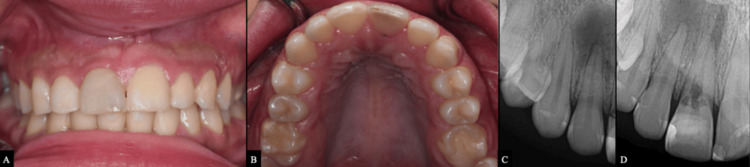
Preoperative clinical photographs and radiographs of tooth #11 showing grayish discoloration. (A) Frontal view. (B) Occlusal view. (C, D) Periapical radiographs demonstrating a periapical radiolucency in the maxillary anterior region and an irregular radiolucent defect in the cervical third of tooth #11.

The patient provided written informed consent for the proposed treatment and publication of her clinical photographs and radiographs. A 20-year-old systemically healthy woman with no known allergies was referred from the restorative clinic for endodontic assessment of a discolored maxillary right central incisor (tooth #11) prior to aesthetic restoration (Figure 1). The patient reported dental trauma to the anterior teeth approximately five years earlier, with no dental consultation at that time. Orthodontic treatment was initiated two years later, after which discoloration of tooth #11 was noted. A composite veneer was subsequently placed to improve aesthetics. However, the discoloration persisted.

Clinical examination

Intraoral examination revealed no swelling or sinus tract in the labial or palatal soft tissues. Tooth #11 had a composite veneer and exhibited grayish discoloration (Figures 1A, 1B). Periodontal probing revealed normal pocket depths, except for tooth #11, which showed bleeding on probing and a 7-mm palatal pocket depth. Percussion and palpation tests were negative.

Pulp sensibility testing was performed using Roeko Endo-Frost cold spray (Coltène/Whaledent, Langenau, Germany). Teeth #11 and #12 showed no response, whereas tooth #13 responded positively. An electric pulp test (EPT) and a test cavity were performed on tooth #12, with no response. The test cavity was used to confirm pulpal status, as cold testing and EPT may be unreliable following orthodontic treatment [11].

CBCT findings

**Figure 2:**
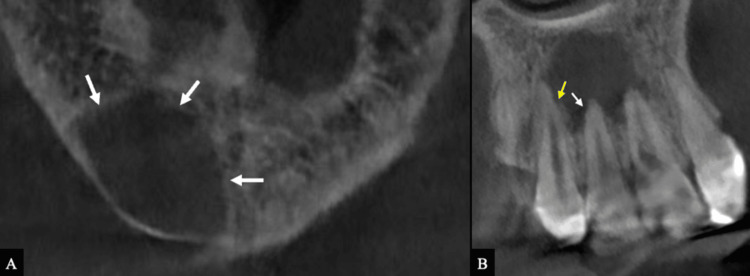
CBCT images of the anterior maxillary region. (A) Axial view demonstrating a large periapical radiolucency with thinning of the labial cortical bone (white arrows). (B) Longitudinal view demonstrating external root resorption in multiple forms, including EIR affecting teeth #11 and #12 (white arrows) and ESR in tooth #13 (yellow arrow). CBCT: Cone beam computed tomography

CBCT imaging revealed a large periapical radiolucent lesion associated with tooth #11, extending distally toward tooth #12, with thinning of the labial cortical bone without expansion or perforation (Figure 2A).

In the longitudinal view, EIR affected the apical third of teeth #11 and #12, while ESR was observed at the apical third of the mesial root surface of tooth #13 (Figure 2B).

**Figure 3:**
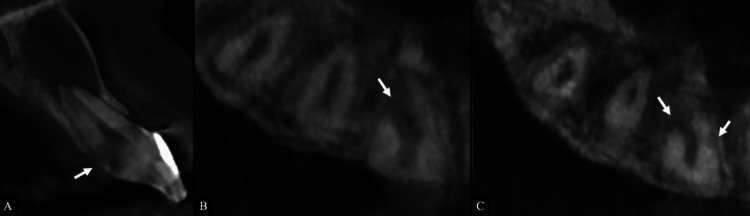
CBCT images of tooth #11 demonstrating ECR. (A) Sagittal view showing a palatal ECR defect extending to the middle third of the root (white arrow). (B, C) Axial views demonstrating circumferential spread ranging from >180° to ≤270°, with evidence of pulpal communication (white arrows). CBCT: Cone beam computed tomography

The sagittal view of tooth #11 demonstrated irregular dentin loss in the cervical region extending toward the middle third of the root (Figure 3A).

Axial views showed a radiolucent resorptive defect in tooth #11 with circumferential spread >180° and evidence of pulpal communication, classified as Class 3Cp according to the Patel et al. ECR classification [12] (Figures 3B, 3C).

Definitive diagnosis

Teeth #11 and #12 were diagnosed with pulp necrosis and asymptomatic apical periodontitis.

Treatment plan and management

Non-Surgical Endodontic Management

Root canal treatment was completed for tooth #12. For tooth #11, an exploratory procedure was performed under a dental operating microscope (DOM; Zumax Medical Co., Jiangsu, China) to assess the feasibility of internal repair. However, failure to achieve adequate hemostasis prevented direct visualization of the defect, thereby precluding internal repair. Accordingly, the canal space was temporarily occupied with a gutta-percha cone without sealer to prevent debris ingress in anticipation of external assessment and repair.

The patient subsequently reported persistent spontaneous pain, with tenderness to percussion and palpation. In conjunction with the presence of a periradicular lesion, surgical intervention was planned to allow enucleation of the pathology. In addition, atraumatic extraction of tooth #11 was planned to permit external evaluation of the ECR defect and the feasibility of IR.

Surgical Endodontic Management

**Figure 4:**
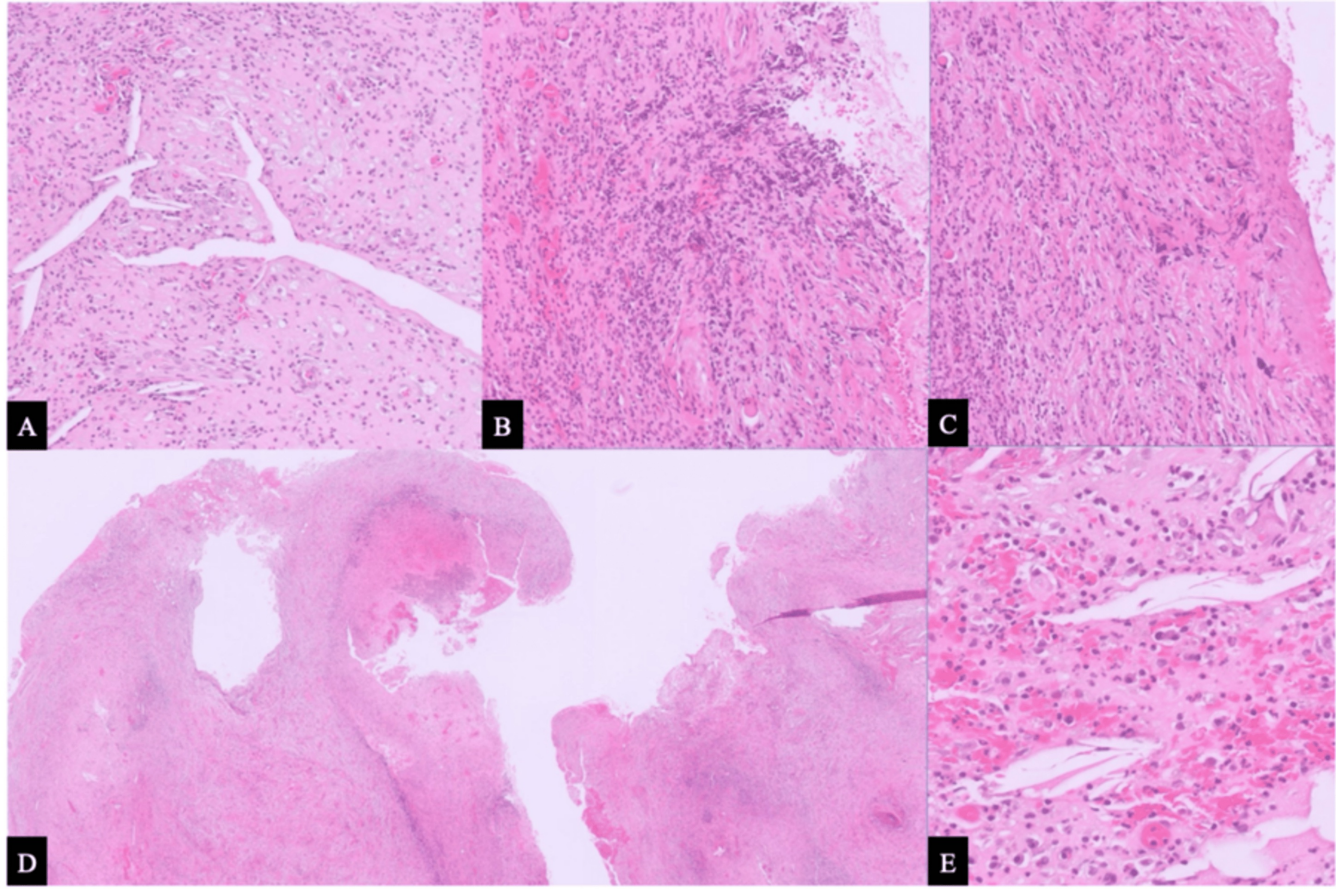
Histopathological features of an inflamed radicular cyst. Hematoxylin and eosin–stained sections show focal ulceration of the epithelial lining of the cyst wall, with dense mixed inflammatory cell infiltration in the underlying connective tissue, along with the presence of cholesterol clefts and granulation tissue. (A–C) Low-power views (×10). (D) Low-power overview (×5). (E) High-power view (×20).

The patient rinsed with 0.12% chlorhexidine gluconate, and local anesthesia was administered using articaine hydrochloride 40 mg/mL with epinephrine 1:100,000. A full-thickness mucoperiosteal trapezoidal flap was reflected using sulcular and vertical releasing incisions. An osteotomy was performed at the site of cortical perforation using a surgical diamond bur under copious saline irrigation. The periradicular lesion was enucleated, and a 3-mm apicectomy was performed on tooth #12. Histopathological examination confirmed an inflamed radicular cyst (Figure 4).

**Figure 5:**
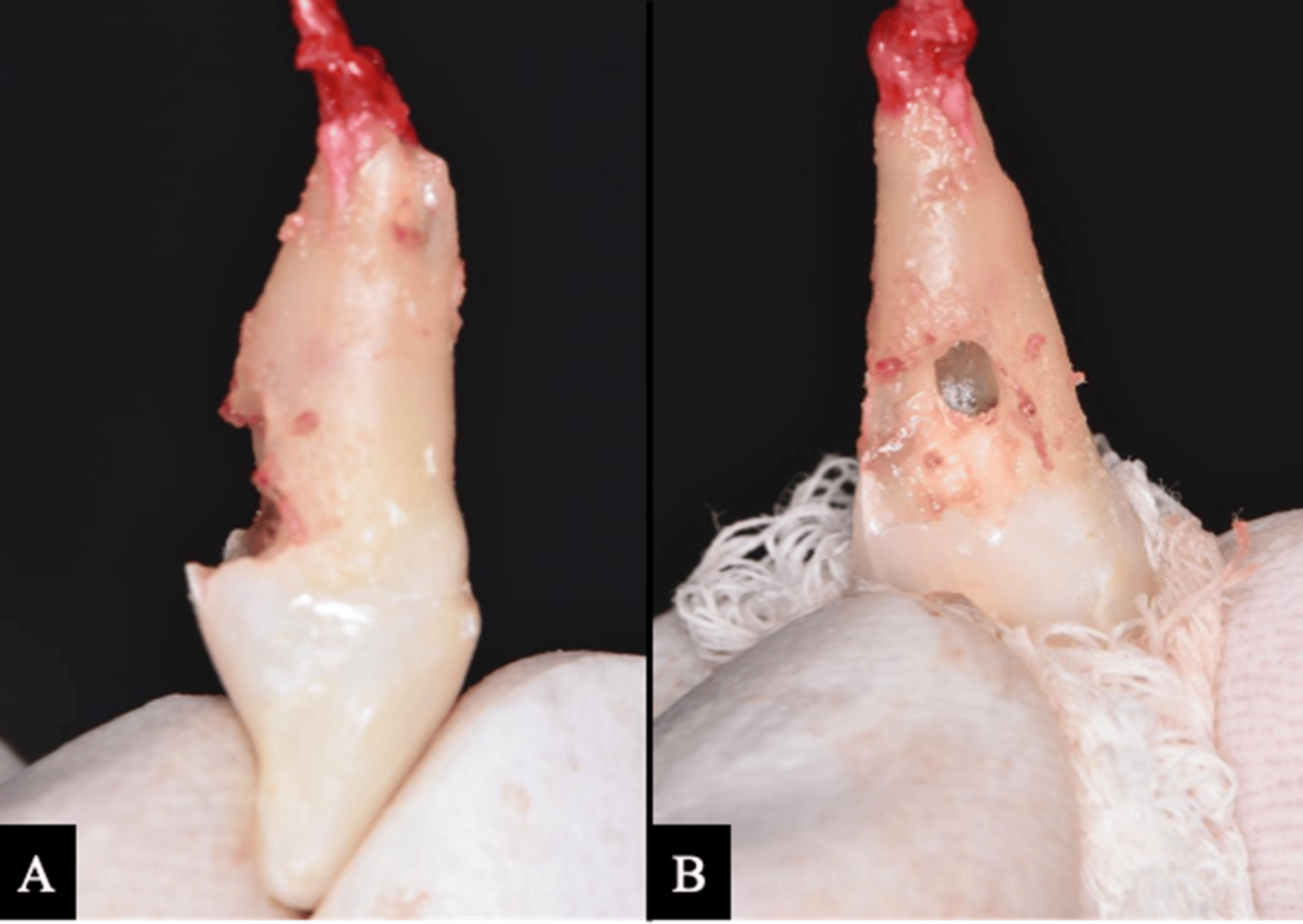
Clinical photographs of tooth #11 after extraction. (A) Lateral view showing multiple resorptive cavities along the root surface. (B) Palatal view showing the resorptive defect causing root undermining extending to the middle third with communication to the pulp space.

Following apicectomy, tooth #11 was extracted using a minimally traumatic technique. The root surface was examined extraorally under a DOM to assess the extent of the resorptive defect. Mechanical debridement revealed significant weakening of the remaining root structure, along with compromised periodontal support (Figure 5). Based on these findings, IR was abandoned in favor of guided bone regeneration (GBR) and socket preservation to facilitate future implant placement.

**Figure 6:**
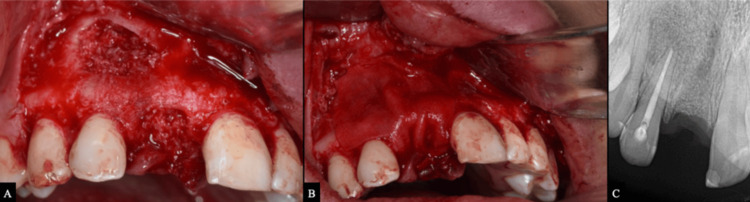
GBR procedure following surgical management. (A) Allograft cancellous bone chips (ReadiGraft®, 0.5 cc; LifeNet Health, Virginia Beach, VA, USA) placed in the bone crypt and the socket. (B) Resorbable collagen membrane (NeoGen® Collagen Flex; Neoss Ltd., Harrogate, UK) applied over the graft. (C) Postoperative periapical radiograph.

GBR was performed to augment the bone crypt and extraction socket using an allograft cancellous bone graft. A resorbable collagen membrane was placed over the graft (Figures 6A, 6B). The flap was repositioned and sutured, and a postoperative periapical radiograph was obtained (Figure 6C).

**Figure 7:**
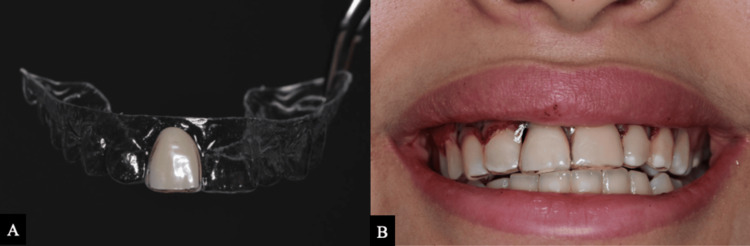
Temporary prosthetic replacement. (A) Essix-type retainer. (B) Appliance in place following extraction of tooth #11.

An Essix-type clear aligner was subsequently provided to maintain aesthetics and preserve the mesiodistal space of the extraction site (Figure 7).

Follow-up

**Figure 8:**
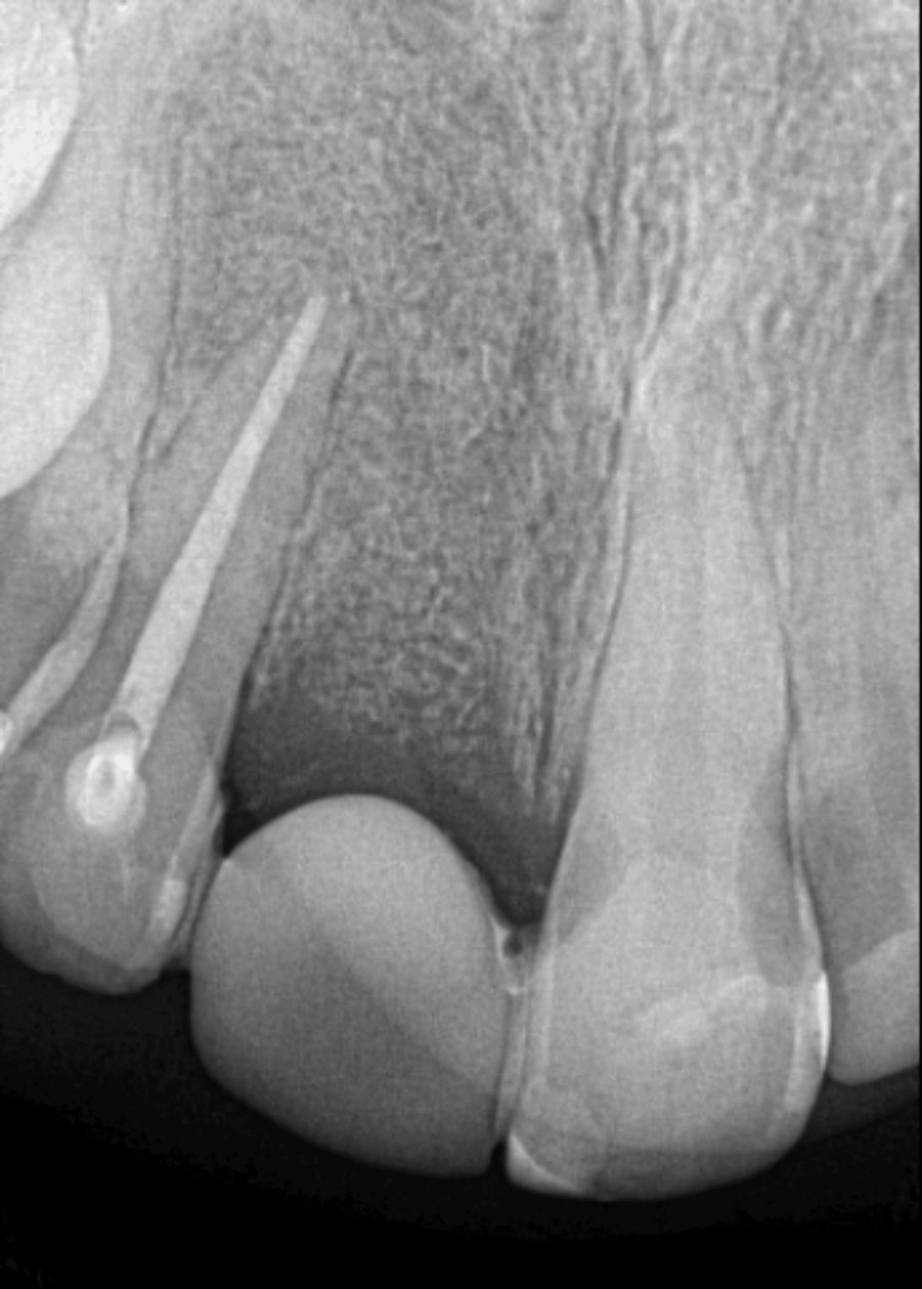
Three-month follow-up. Periapical radiograph showing no evidence of periapical radiolucency and signs of bone healing.

At the three-month follow-up, clinical and radiographic examinations revealed no abnormal findings (Figure 8). The patient was referred for implant placement. Long-term clinical and radiographic follow-up will be performed at regular intervals to monitor bone healing and the pulp and periapical status of teeth #12 and #13.

## Discussion

In the present case, the observed forms of ERR were likely triggered by a history of dental trauma followed by orthodontic treatment [3]. The ESR on tooth #13 was associated with localized factors, including pressure from adjacent pathology [3]. Clinical findings, including a positive response to cold testing, confirmed normal pulp vitality and a non-infective resorptive process, distinguishing it from EIR [1,6]. ECR on tooth #11 was located on the palatal surface of the maxillary central incisor, which is the tooth most frequently affected by resorption [7].

The ECR on tooth #11 was classified as Patel Class 3Cp, indicating extension into the middle third of the root, circumferential spread >180° to ≤270°, and pulpal involvement [12]. These features made internal repair challenging, as complete visualization and hemostasis were not achievable during the non-surgical phase. Furthermore, an external surgical approach was avoided because palatal and interproximal ECR defects often present with limited visibility and accessibility, making debridement difficult [10].

Extraoral repair via IR was considered as a final attempt to preserve the natural tooth [10,13]. However, this approach was abandoned because of significant structural and periodontal concerns. Debridement of the resorptive tissue resulted in a weakened remaining root structure, predisposing it to fracture [14]. Additionally, successful replantation relies on preservation of a viable PDL [10]. However, in ECR cases, the PDL and protective cementum are frequently damaged or absent in the cervical region, increasing the risk of ankylosis [10,15]. Furthermore, long-term clinical evidence indicates that the odds of a poor prognosis are more than three times higher in cases involving pulpal involvement, palatal localization, or multiple affected surfaces [9].

Given these considerations, a single-tooth implant was selected due to its more favorable long-term prognosis, which has been shown to exceed 90% and is significantly higher than that reported for IR [16]. Due to a significant loss of alveolar bone, GBR and socket preservation were indicated. These procedures maintain alveolar ridge width by excluding proliferating soft tissue cells, thereby creating an optimal environment for bone formation and supporting periradicular healing prior to implant placement [17].

Postoperative swelling developed after the procedure and was managed with a 4 mg intramuscular injection of dexamethasone, with resolution by the following day [18]. Such flare-ups have been reported in 1.4-16% of cases and are more common in female patients younger than 20 years, particularly in those with large periapical lesions [19,20].

## Conclusions

This case highlights the synergistic effect of dental trauma and orthodontic treatment in the development of external root resorption (ERR). External cervical resorption (ECR) is an aggressive and progressive condition that may remain clinically silent until the patient presents with tooth discoloration, often associated with pulpal communication and the development of a radicular cyst.

Given its destructive nature, management requires a multidisciplinary approach. Careful clinical and radiographic assessment is essential, and in patients with a history of dental trauma, CBCT prior to orthodontic treatment, along with close monitoring during treatment, may aid in early detection and limit disease progression.

## References

[1] Bauss O, Röhling J, Meyer K, Kiliaridis S (2009). Pulp vitality in teeth suffering trauma during orthodontic therapy. Angle Orthod.

[2] Chen Y, Huang Y, Deng X (2021). External cervical resorption-a review of pathogenesis and potential predisposing factors. Int J Oral Sci.

[3] Patel S, Krastl G, Weiger R (2023). ESE position statement on root resorption. Int Endod J.

[4] Patel S, Mavridou AM, Lambrechts P, Saberi N (2018). External cervical resorption-part 1: histopathology, distribution and presentation. Int Endod J.

[5] Neuvald L, Consolaro A (2000). Cementoenamel junction: microscopic analysis and external cervical resorption. J Endod.

[6] Mavridou AM, Hauben E, Wevers M (2016). Understanding external cervical resorption in vital teeth. J Endod.

[7] Patel S, Abella F, Patel K, Lambrechts P, Al-Nuaimi N (2023). Clinical and radiographic features of external cervical resorption - an observational study. Int Endod J.

[8] Irinakis E, Aleksejuniene J, Shen Y, Haapasalo M (2020). External cervical resorption: a retrospective case-control study. J Endod.

[9] Suhr Villefrance J, Kirkevang LL, Wenzel A (2024). Long-term prognosis for teeth with external cervical resorption based on periapical images and cone beam CT: A clinical study. Int Endod J.

[10] Patel S, Foschi F, Condon R (2018). External cervical resorption: part 2 - management. Int Endod J.

[11] Nangia D, Duggal I, Logani A (2025). Reliability of electric pulp test and thermal pulp test for assessing pulpal response in patients undergoing orthodontic treatment - a systematic review. Int Orthod.

[12] Patel S, Foschi F, Mannocci F, Patel K (2018). External cervical resorption: a three-dimensional classification. Int Endod J.

[13] Machado IC, Morais MO, Bicalho AL (2024). Prevalence and characterization of external cervical resorption using cone beam computed tomography. J Endod.

[14] Manaktala M, Taneja S, Bhalla VK (2024). Stress distribution in endodontically treated external cervical resorption lesions restored with MTA and biodentine - a finite element analysis. J Oral Biol Craniofac Res.

[15] Cho SY, Lee Y, Shin SJ (2016). Retention and healing outcomes after intentional replantation. J Endod.

[16] Torabinejad M, Dinsbach NA, Turman M (2015). Survival of intentionally replanted teeth and implant-supported single crowns: a systematic review. J Endod.

[17] Flynn R, Foschi F, Maloney B (2024). The impact of bone grafting with/without barrier membrane placement on the outcome of apical surgery: a systematic review and meta-analysis. Int Endod J.

[18] Lin S, Levin L, Emodi O (2006). Etodolac versus dexamethasone effect in reduction of postoperative symptoms following surgical endodontic treatment: a double-blind study. Oral Surg Oral Med Oral Pathol Oral Radiol Endod.

[19] Siqueira JF Jr (2003). Microbial causes of endodontic flare-ups. Int Endod J.

[20] Alghamdi A, Mominkhan D, Sabano R (2025). Factors associated with postsurgical pain and swelling following endodontic microsurgery: the role of radiographic characteristics. Healthcare (Basel).

